# Evaluations of Sex Differences in Dosimetry in Rats Following Perfluorohexanesulfonamide (PFHxSA) Oral Exposure

**DOI:** 10.3390/toxics13121022

**Published:** 2025-11-26

**Authors:** Jackson G. Bounds, Aero Renyer, Jermaine L. Ford, Krishna Ravindra, Michael J. Devito, Michael F. Hughes, Leah C. Wehmas, Amanda A. Brennan, Barbara A. Wetmore, Denise K. MacMillan

**Affiliations:** 1 Oak Ridge Associated Universities (ORAU), Oak Ridge, TN 37830, USA; 2 Oak Ridge Institute for Science and Education (ORISE), Oak Ridge, TN 37830, USA; 3 Center for Computational Toxicology and Exposure, Office of Research and Development, U.S. Environmental Protection Agency (USEPA), Durham, NC 27709, USA; ford.jermaine@epa.gov (J.L.F.); devito.michael@epa.gov (M.J.D.); wetmore.barbara@epa.gov (B.A.W.)

**Keywords:** PFAS, perfluoroalkyl sulfonamide, PFHxS, PFASA, LC/MS/MS, thyroid hormone disruption, hepatocyte clearance, renal clearance, IVIVE

## Abstract

Perfluorohexanesulfonamide (PFHxSA) is used as a replacement for legacy PFAS. Non-targeted analysis identified perfluorohexane sulfonic acid (PFHxS) as the primary metabolite of PFHxSA in plasma and liver in a short-term (5-day) repeat dose study with male and female Sprague Dawley rats (Crl:CD(SD)). This evaluation sought to quantitate PFHxSA and PFHxS concentrations by targeted liquid chromatography/mass spectrometry (LC/MS/MS) to further evaluate metabolism and dosimetry following in vivo PFHxSA exposure. In males, quantified plasma and liver PFHxS concentrations were higher than those of its parent, PFHxSA. PFHxS was detected in female plasma and liver at on average 5.3- and 2.9-fold lower, respectively, than PFHxSA. In both sexes, plasma and liver PFHxSA dose concentrations decreased with increasing doses, suggesting hepatic enzyme induction. Liver-to-plasma partitioning favored plasma across all doses in both sexes. In vitro–in vivo extrapolation (IVIVE) suggests higher steady-state plasma concentrations in humans vs. rats for PFHxSA and PFHxS. The in vivo concentrations aligned reasonably (i.e., within 6- to 12.1-fold) with the IVIVE-derived rat plasma estimates. Identifying when PFAS co-exposures may result due to metabolic biotransformation of the parent PFAS to a stable and potentially bioactive metabolite is important to better inform the interpretation of in vivo and in vitro findings.

## 1. Introduction

Per- and polyfluoroalkyl substances (PFAS) are a class of compounds used extensively in industry and consumer goods due to their advantageous amphipathic properties and the stability of their carbon–fluorine (C-F) bonds [1]. Initially, PFAS production was dominated by compounds with a longer C-F backbone, such as perfluorooctanoic acid (PFOA) and perflurooctanesulfonic acid (PFOS); however, these legacy PFASs were phased out in the United States and Canada and added to the Stockholm Convention after adverse toxicological effects were proposed in humans and the environment [2,3,4,5,6,7]. Currently, alternatives to legacy PFASs are being developed, collectively referred to as emerging PFAS, which may differ in terms of C-F chain length (generally ≤6 carbons), bond linkages, and head groups [8,9]. Emerging PFAS are often presumed to be less toxic and quicker to degrade [10]. Nevertheless, the recent literature suggests that emerging PFASs are environmentally persistent and potentially as or more toxic than legacy PFAS [11,12,13,14,15,16,17].

**Figure 1:**
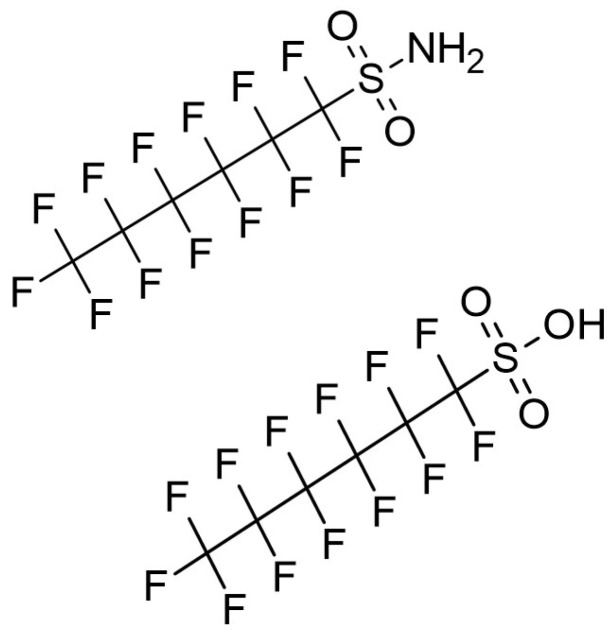
Chemical structures of perfluorohexanesulfonamide (PFHxSA) (**top**) and perfluorohexane sulfonic acid (PFHxS) (**bottom**).

Perfluoroalkyl sulfonamides (PFASAs) are a subclass of emerging PFAS comprising a fluorinated carbon chain with a sulfonamide head group. PFASAs have multiple industrial uses, including as substitutes for the legacy compound PFOS in aqueous film-forming foams (AFFFs) employed in firefighting applications [18,19,20]. Perfluorohexanesulfonamide (PFHxSA) (Figure 1) has been detected at AFFF-contaminated sites [19,21,22,23,24,25] as well as surrounding soil, surface water, ground water, and associated biota [19,21,22,23,24,25,26,27,28]. Hydrolysis of PFAS common in AFFF during water treatment was shown to yield PFHxSA [29], and six-carbon sulfonamido precursors were recently shown to be six times more abundant than perfluorohexane sulfonic acid (PFHxS) (Figure 1) in AFFF produced by 3M [19]. Unlike perfluoroalkyl sulfonic acids (PFSAs), PFHxSA, other PFASAs, and their sulfonamido precursors are seldom included in targeted mass spectrometry methods for environmental samples, therefore hampering our understanding of potential environmental and human exposure.

Potential health effects from exposure to PFASAs are beginning to appear in the scientific literature. The majority of the studies are in non-mammalian species. Perfluorobutanesulfonamide (PFBSA) was shown to perturb zebrafish embryo morphology and yield higher tissue concentrations than other short-chain PFAS [30]. Recent studies demonstrated that PFHxSA was a more potent developmental toxicant than PFOS, itself a significant developmental toxicant in amphibian and zebrafish embryos [31,32]. Another study found sex-dependent behavioral aberrations that may also have been maternally transferred to the first-generation offspring in zebrafish exposed to PFHxSA [33]. Several toxicological studies have been published for perfluorooctanesulfonamide (PFOSA) detailing adverse effects such as cardiotoxicity, developmental toxicity, and hepatotoxicity in fish and developmental toxicity in nematodes [34,35,36,37,38,39]. In rats, Auerbach et al. [40] observed thyroid hormone (TH) disruption, liver and body weight changes, and transcriptomic changes following exposure to PFHxSA. The limited number of mammalian studies with this class of PFAS warrants further toxicological study.

In addition to reports of toxicity to multiple species, PFASAs are known precursors to PFSAs and perfluorocarboxylic acids (PFCAs) [23,41,42,43,44,45]. As examples, PFOSA is metabolized to PFOS [46], and PFHxSA is biotransformed to PFHxS [45,46]. Both biotransformation products (BPs) have been shown to be toxic to multiple species [47,48,49,50], and both are of concern to the United States Environmental Protection Agency (US EPA) [51,52]. Uses of PFOS and PFHxS- and PFHxS-related compounds were restricted by addition to the Stockholm Convention of Persistent Organics Pollutants in 2009 and 2022, respectively [5]. As PFHxSA and its precursors have been observed in the environment and all may be precursors to the known toxicant PFHxS, an evaluation of disposition and effects from exposure to PFHxSA in mammals is warranted [19,21,22,23,24,25].

This research effort sought to characterize PFAS exposures, dosimetry, and thyroid effects following in vivo PFHxSA (Figure 1) administration to Sprague Dawley (SD) rats. Plasma and liver were obtained from a companion study, a recent United States (US) Environmental Protection Agency Transcriptomic Assessment Product (ETAP) [53,54] for PFHxSA [55], wherein transcriptomic points of departure were derived for benchmark dose estimation. In a recent non-targeted analysis (NTA) of plasma and liver from the same study, PFHxS was noted as the major metabolite, although levels were not quantified [45]. Consequently, these evaluations aimed to quantify PFHxSA and PFHxS in rat liver and plasma to further elucidate metabolism, systemic exposures, disposition, and sex differences [40,45,55]. In vitro toxicokinetics data in rat and human hepatocytes and plasma were also generated to evaluate in vitro–in vivo extrapolation approaches and cross-species comparisons.

## 2. Materials and Methods

### 2.1. In Vivo Study Design

The plasma and liver samples for this investigation were provided by Mutlu et al. [55]. The study design, including detailed descriptions of animal handling, procedures for dosing, clinical observations, termination, and collection of tissues, is reported elsewhere [45,53,54,55] and is consistent with the ARRIVE guidelines. Briefly, male and female Sprague Dawley (SD) rats (Crl:CD(SD)) (*n* = 48/sex, 96 total) were purchased from Charles River Laboratory (Raleigh, NC, USA) and allowed to acclimate for ≥5 days before initial dosing. Rats were 8–10 weeks old at the time of exposure. PFHxSA dosing solutions in corn oil were prepared on Day 0 and used over the course of the study. Rats of both sexes (*n* = 5/sex, dose, and time point, plus eight vehicle controls per sex) were dosed by oral gavage (5 mL/kg body weight) once daily for five days. As detailed fully in Mutlu et al. [55], at the time of study initiation, PFHxSA did not have any existing acute or repeat dose toxicity studies to assist in identifying the potential dose range. The high dose was set at 100 mg/kg/day (250 µmol/kg/day) based on the maximum solubility achieved in corn oil as well as previous studies by Auerbach et al. [40] that observed mortality in a 5-day study of PFHxSA at exposures above 100 mg/kg/d. The formulation concentrations decreased from 100 mg/kg/day at half-log_10_ intervals until the lowest dose level, which was a full log_10_ interval below the next higher dose, giving eight dose levels (0.01 to 100 mg/kg/day or 0.025 µmol/kg/day to 250 µmol/kg/day) in addition to vehicle controls. Doses were administered in randomized order within ± 1 h on each day of dosing. All rats survived to termination. Rats were euthanized 24 h after the last dose. Blood was collected via cardiac puncture with tripotassium ethylene diamine tetraacetic acid (K_3_EDTA) as an anti-coagulant and centrifuged to collect cell-free plasma. Liver was collected and flash-frozen in liquid nitrogen. Liver, plasma, and dosing formulation samples were frozen at the USEPA Center for Computational Toxicology and Exposure (CCTE), Durham, NC, USA. Tissue samples (plasma and liver, *n* = 96 each) were stored at ≤−70 °C prior to extraction; dosing formulation samples (*n* = 9) were stored at −20 °C.

### 2.2. Analytical Chemistry

#### 2.2.1. Chemical Information, Dosing Formulation Determination, and Stability Assessment

Perfluorohexanesulfonamide (PFHxSA; CAS 41997-13-1; DTXSID50469320) [5] was purchased from Synquest Laboratories (Alachua, FL, USA; purity > 99%). Corn oil was used as the dosing vehicle and purchased from Welch, Holme & Clark Co. (Newark, NJ, USA). Prior to dose administration, the purity of PFHxSA was confirmed to be approximately 99.8% by RTI International (Durham, NC, USA; Contract No.: 68HERC21D0004) using gas chromatography/mass spectrometry (GC/MS). Neither PFOS nor PFOA was detected above the method detection limit (5 ppm).

Information on chemicals and standards used may be found in Supplementary Information (SI) Text S1 and Table S1. Refer to SI Text S2 for the dosing solution extraction method and information on calibration and QC parameters. Samples were extracted in a batch together with a method blank (MB), continuing calibration verification (CCV), and a laboratory control sample (LCS) for quality control (QC). QC samples were prepared in laboratory-grade corn oil (Sigma Aldrich; St. Louis, MO, USA). Extracts were analyzed on a Sciex 4000 QTRAP liquid chromatography/tandem mass spectrometer (LC/MS/MS) as described below for PFHxSA in plasma and liver (see Section 2.2.3). The limit of quantitation (LOQ) for PFHxSA in the dosing-solution extract was 50 ng/mL.

The stability of PFHxSA corn oil dosing solutions was also evaluated up to 14 days. PFHxSA solutions were prepared using the same lot number of the chemical as was used in Mutlu et al. [55]. Solutions were prepared under an inert atmosphere at a 20,000 µg/mL (20 mg/mL) high-dose solution and a 2 µg/mL (0.002 mg/L) low-dose solution to mimic the range of concentrations of the dosing formulations used for the in vivo study. Each batch included an MB and an LCS prepared from blank corn oil as QC. The solutions were split into two aliquots each. One aliquot was stored at 4 °C; the other aliquot was stored at room temperature, with both protected from light. The stability samples were analyzed in triplicate on Days 0, 5, 7, and 14 using the same extraction method and LC/MS/MS conditions outlined in SI Texts S2 and S3, respectively. Stability was assessed by using mean peak areas to calculate the percent recovery for each day of analysis relative to Day 0. Information on percent recovery calculation and additional experimental details concerning the stability study may be found in SI Text S2.

#### 2.2.2. Plasma Thyroid Hormone Quantitation

Thyroid hormones (TH) triiodothyronine (T3), reverse triiodothyronine (rT3), and thyroxine (T4) (SI Table S1) were extracted from plasma spiked with ^13^C-labeled TH in batches of up to 96 samples, along with an MB, CCV, and LCS for QC. Gibco (Billings, MT, USA) Dulbecco’s Phosphate-Buffered Saline was used as the control matrix. See SI Text S3 for the extraction procedure.

Samples were analyzed as previously described in O’Shaughnessy et al. [56]. Isotope dilution targeted LC/MS/MS was performed using a Sciex (Framingham, MA, USA) 6500+ QTRAP liquid chromatography/linear ion trap mass spectrometer system operated in positive electrospray ionization (ESI) mode with multiple reaction monitoring (MRM) scanning. A Restek (Bellefonte, PA, USA) Raptor Biphenyl column (100 mm × 2.1 mm, 2.6 µm) was used for chromatographic separation with a flow rate of 0.3 mL/min and a 50 °C column temperature. Gradient elution (see SI Table S2) was obtained with 0.1% formic acid in water (H_2_O) (mobile phase A) and 0.1% formic acid in methanol (MeOH) (mobile phase B). Detection limits for T3, rT3, and T4 were 0.04 ng/mL in plasma.

Additional instrument parameters can be found in SI Table S3; MRM transitions are listed in SI Table S4. Calibration and QC parameters for TH analysis are found in SI Text S4. Statistical analysis was performed as described in SI Text S5.

#### 2.2.3. Plasma and Liver Dosimetry

Detailed information on solvents and standards used for targeted LC/MS/MS of PFHxSA and PFHxS can be found in SI Text S1 and SI Table S1. Plasma and liver preparations were modified from Conley et al. [57]. Briefly, aliquots of plasma (25 µL) and liver (~10 mg) were spiked with PFOSA as an internal standard, as no isotope-labeled PFHxSA was available. Sample batches included up to 96 samples along with an MB, CCV, and LCS for QC. Commercial plasma (mixed-sex SD rat plasma) and liver (mixed-sex SD rat) were obtained from BioIVT (Westbury, NY, USA). Cold acetonitrile (ACN) with 0.1 M formic acid was used to precipitate proteins. Liver and plasma samples were prepared similarly, except that the livers were homogenized after solvent addition. Plasma samples were vortexed to mix before being chilled at −20 °C, after which samples were centrifuged at 5 °C and 10,000× *g*. Liver samples were chilled at −80 °C, then homogenized using an Omni International (Kennesaw, GA, USA) Bead Ruptor 24. Liver homogenates were centrifuged at 26,000× *g* and 5 °C. Supernatants from the extraction of both matrices were removed and stored at −20 °C until analysis. Refer to SI Text S4 for additional experimental details and information on calibration and QC parameters for quantitation of PFHxSA.

Targeted LC/MS/MS of PFHxSA was performed on a Sciex 4000 QTRAP LC/MS/MS system operated in negative ESI mode with MRM scanning. A Restek (Bellefonte, PA, USA) Raptor Polar X (100 mm × 2 mm, 2.7 µm) column was used for liquid chromatography. Isocratic elution occurred over 10 min with a 30:70 ratio of mobile phase A (10 mM ammonium formate): mobile phase B (60:40 ACN: methanol). Both mobile phases included 0.05% formic acid. The LOQs for PFHxSA were 0.100 µM in plasma and 0.351 µM in liver. Additional details of the instrument method parameters for targeted analysis of PFHxSA are provided in SI Table S5. The MRM transitions for the PFHxSA method are provided in SI Table S6.

For the determination of PFHxS in plasma and liver, an extraction protocol identical to that used for PFHxSA was employed, except that labeled PFHxS (^13^C_3_-PFHxS, >98% purity) from Wellington Laboratories (Guelph, ON, CA) was used as an internal standard for isotope dilution. MRM transitions for PFHxS are provided in SI Table S6. LC separation of PFHxS was conducted as follows: a Phenomenex (Torrance, CA, USA) Kinetex EVO C18 (100 mm × 2.1 mm, 2.6 µm) analytical column was used with a gradient outlined in SI Table S7. Mobile phase A was 95:5 H_2_O:MeOH; mobile phase B was 50:50 MeOH:ACN. Both mobile phases also contained 10 mM ammonium acetate. LOQs for PFHxS were 1.38 µM in plasma and 1.73 µM in liver. For those samples with PFHxS and PFHxSA concentrations below the LOQ, concentrations were estimated based on half the LOQ only for dose levels in which four rats (per sex) had concentrations above the LOQ; all other samples were non-detect. Refer to SI Text S5 for information on significance testing and calculation of percent dose.

### 2.3. In Vitro Hepatocyte Metabolic Stability Assay Materials, Chemicals, and Extraction

In vitro evaluations of PFHxSA and PFHxS hepatocyte metabolic stability were performed in rat hepatocytes, as previously described [58]. Also, human hepatocyte clearance data were obtained from an earlier study [58]. Native PFHxSA was obtained from Alpha Chemistry (purity > 98.4%; Holbrook, NY, USA), and PFHxS was obtained from Ark Pharma Scientific Limited (purity > 95%; Cambridge, UK). Pooled, mixed-sex cryopreserved hepatocyte suspensions were employed in a substrate depletion approach, using a final assay concentration of 1 μM. At each time point (0, 15, 30, 60, 120, 240 min), assay samples were crashed with an equal volume of ice-cold ACN containing 1.2% formic acid, and relevant internal standard (M3PFHxS for PFHxS; M8PFOS for PFHxSA; >98% purity; Wellington). After chilling and centrifugation at 4200× *g*, the supernatants were transferred to new collection plates and stored at <−70 °C until analysis for the parent compounds on the Waters instrumentation described below. Immediately prior to analysis, samples were thawed, vortexed, and centrifuged at 4200× *g*, and then diluted 1:4 in 95:5 water/ACN with 2.5 mM of ammonium acetate. Negative controls, including no cell controls and metabolically inactivated hepatocytes, were run concurrently to assess the chemical stability over the time course; reference compounds, propranolol and/or phenacetin, were run concurrently to assess the assay performance.

### 2.4. Ultracentrifugation Plasma Protein Binding Assay Chemicals, Materials, Study Design, and Calculations

Human and rat plasma were evaluated for plasma protein binding of PFHxSA and PFHxS using ultracentrifugation. Data generated in human plasma were obtained from an earlier study [59]; for this study, data were generated in rat plasma following the same protocol as described by Smeltz et al. [59]. SD rat plasma (pooled, mixed sex) was obtained from a commercial vendor (BioIVT, Westbury, NY, USA). Briefly, PFHxSA and PFHxS were added to the plasma to achieve a final assay concentration of 10 µM. Following a 1 h 37 °C incubation, 1 mL aliquots from each plasma solution were drawn in triplicate and transferred to polycarbonate ultracentrifuge tubes. These samples underwent ultracentrifugation (Beckman OptimaMax; Beckman Coulter Inc., Indianapolis, IN, USA) at 850,000× *g* for 4 h at 37 °C; after completion, the aqueous fraction (AF) was collected by transferring the supernatant to a new tube. Two sets of samples of the original PFAS-spiked whole plasma were also collected after a 1 h or a 5 h incubation at 37 °C. All samples were combined with 3 volumes of ice-cold ACN containing relevant internal standard, vigorously mixed, then centrifuged at 12,000× *g* for 10 min at 4 °C. Supernatants were collected and then stored at <−70 °C until analysis.

### 2.5. In Vitro Study Targeted Sample Analysis

Targeted, quantitative analysis of in vitro hepatocyte metabolic stability and plasma protein binding assay samples was conducted on a Waters (Milford, MA, USA) Xevo-TQS micro ultra-high-performance liquid chromatography tandem mass spectrometry system (UPLC-MS/MS), modified with a PFAS analysis kit. Samples were analyzed in negative ion mode with UniSpray ionization. Samples were analyzed in triplicate, with a reference compound included to evaluate assay performance. A CORTECS T3 column (Waters; 3 mm × 100 mm, 2.7 µm) was used for chromatographic separation with a flow rate of 0.6 mL/min and a temperature of 55 °C. Gradient elution was used with mobile phase A (95:5 H_2_O/ACN) and mobile phase B (95:5 ACN/H_2_O), each with 2.5 mM of ammonium acetate added. More details are provided in Smeltz et al. [59].

### 2.6. Toxicokinetic Data Analysis and In Vitro-In Vivo Extrapolation to Estimate C_ss_

Intrinsic hepatic clearance (Cl_int_) and fraction unbound in plasma (f_up_) were calculated from the experimental point estimate data as described in Dobreniecki et al. [60]. Steady-state concentrations (C_ss_) were derived based on zero-order uptake of an assumed daily dose from the gut (assuming 100% intestinal bioavailability) but considering hepatic clearance and non-metabolic renal clearance [61]. Renal clearance was calculated using the glomerular filtration rate (a species-dependent constant) and the fraction unbound in blood (f_ub_), estimated by adjusting the experimental f_up_ for blood/plasma partitioning. The C_ss_ calculations, along with underlying species-specific physiologic scalars, are detailed in SI Texts S6 and S7.

### 2.7. Statistical Analysis

Data were evaluated for normality based on the Kolmogorov–Smirnov test. Data that failed normality tests were then log-normalized. Two-way ANOVAs followed by Bonferroni’s multiple comparison test were performed using GraphPad Prism version 8.3.0 (GraphPad Software, LLC; Boston, MA, USA). Comparisons between males and females were performed only on dose levels where at least 3 out of 5 animals had measured values above the LOQ. In these cases, one-half of the LOQ was used to estimate the missing data. The percent dose/gram tissue (%dose/g tissue) data were fit to the following equation using GraphPad Prism version 8.3.0 (GraphPad Software, LLC; Boston, MA, USA):

$$Y=bottom+\frac{Top-Bottom}{1+(\frac{X}{EC50})}$$

An extra sum of squares F test was performed to determine if the curve adequately fits both data sets.

## 3. Results

### 3.1. Concentrations and Stability of Dosing Formulations

The concentrations of PFHxSA in the dosing formulations used for the in vivo study were confirmed to be within 10% of the expected value, except for the 6000 µg/mL (6 mg/mL) solution, which gave an observed concentration that was +34% from the nominal concentration. No PFHxSA was detected in the vehicle controls or MB. The PFHxSA concentrations observed for the individual dosing formulations are presented in SI Table S8.

PFHxSA was soluble in corn oil at concentrations of 20,000 µg/mL (20 mg/mL). Stability studies indicated that solutions between 2 µg/mL (0.002 mg/mL) and 20,000 µg/mL (20 mg/mL) were stable (within ±30% of the initial value) for up to 14 days at room and refrigerated (4 °C) temperatures. See SI Table S9 for detailed results.

### 3.2. Thyroid Hormone Concentrations

Concentrations of T4 generally decreased with increasing dose (Table 1). Two-way ANOVA testing indicated that dose and sex had a significant impact on T4 plasma concentrations (*p* < 0.05) and indicated statistically significant interaction between the variables compared to controls for males (10, 30, 100 mg/kg/day) (*p* < 0.0001) and females (100 mg/kg/day) (*p* < 0.001). For male and female rats, T3 concentrations were statistically different from controls only at the 100 mg/kg/day dose (*p* < 0.05). Two-way ANOVA testing indicated that dose and sex had a significant impact on T3 plasma (*p* < 0.05), but no significant interactions were noted between dose and sex. rT3 was not detected in female rat plasma and sporadically in male rats with no dose-responsive effect (SI Table S10). Data for individual rats may be found in SI Table S10.

### 3.3. PFHxSA and PFHxS in Plasma

**Figure 2:**
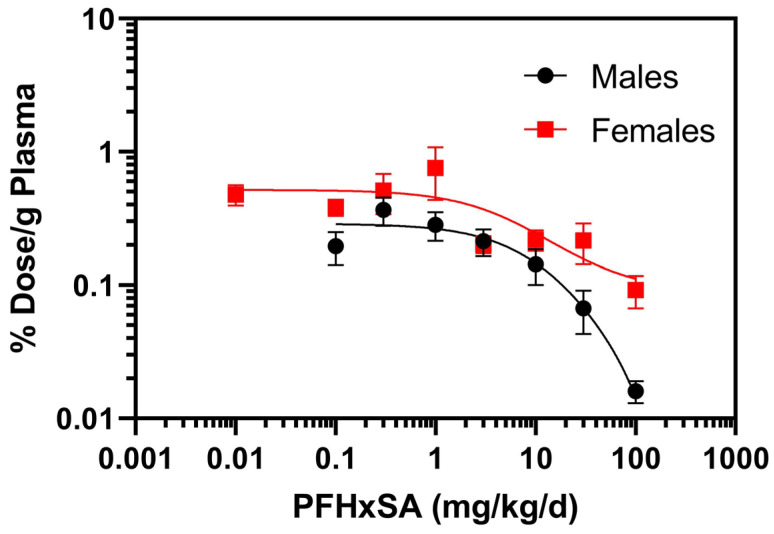
Normalized PFHxSA concentrations to percent (%) dose per gram (g) of plasma after five days of exposure. Data points denote mean ± SD (*n* = 5). Note: for the 0.01 mg/kg/day cohort, results for four of the male rats and one female rat were below the LOQ. Data for the one male with quantifiable PFHxSA at the 0.01 mg/kg/day cohort was not included.

Individual PFHxSA rat plasma data may be found in SI Table S11. Female rat plasma PFHxSA concentrations were similar to those measured in male rats at doses below 30 mg/kg/day (Table 2). At 30 and 100 mg/kg/day, female PFHxSA plasma concentrations were at least two-fold higher than in male plasma. Two-way ANOVA testing indicated that dose and sex had a significant impact on PFHxSA plasma concentrations (*p* < 0.0001) and indicated a statistically significant interaction between the variables (*p* < 0.0001). Normalization of PFHxSA concentrations to percent dose per gram of plasma revealed a dose-dependent decrease in % dose/g plasma (Figure 2). Extra sum of squares F test (DFn = 12.15 DFd (3,9) indicated that the dose–response relationships were different between males and females (*p* < 0.0001).

PFHxS plasma samples are presented in Table 3. A significant difference was observed in the plasma concentrations of PFHxS between sexes (*p* < 0.0001) at all doses tested. Male plasma exhibited 4–10 times higher PFHxS concentrations in plasma compared to the concentrations in female plasma at the same dose level across the dose range. Overall, two-way ANOVA testing indicated that both dose and sex were significant factors in the trends observed for PFHxS plasma concentrations (*p* < 0.0001, both factors), as was the interaction between dose and sex (*p* = 0.0002). Individual rat PFHxS plasma data may be found in SI Table S12.

### 3.4. PFHxSA and PFHxS in Liver

The mean PFHxSA liver concentrations in male rats ranged from 5.41 µM for the 0.1 mg/kg/day dose level to 265 µM for the high dose level (100 mg/kg/day) (Table 4). Mean PFHxSA concentrations in the liver for the female cohort ranged from 4.80 µM at the 0.1 mg/kg/day dose to 594 µM at the highest dose (Table 4). As was observed for plasma, the results of the two-way ANOVA of the liver indicated that results for both dose and sex (*p* < 0.0001) as well as the interaction of the two were significant (*p* = 0.0092). Liver concentrations of PFHxSA were significantly higher in females compared to males at the 30 mg/kg/day dose (*p* < 0.03). Individual rat liver PFHxSA concentrations may be found in SI Table S13.

**Figure 3:**
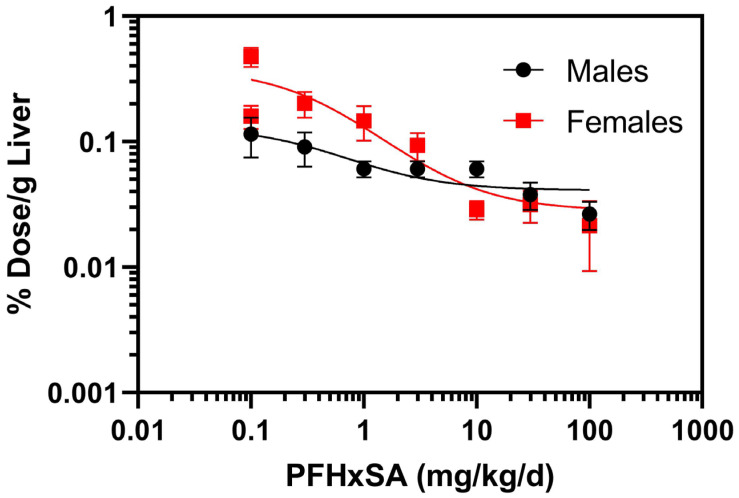
Normalized PFHxSA concentrations to percent (%) dose per gram (g) of liver after five days of exposure. Data points denote mean ± SD (*n* = 5).

Normalization of liver PFHxSA concentrations to percent dose per gram of liver (% dose/g liver) revealed a dose-dependent decrease in % dose/g liver (Figure 3). Extra sum of squares F test (DFn = 27.8 DFd (3,64) indicated that the dose–response relationships were different between males and females (*p* < 0.0001).

**Figure 4:**
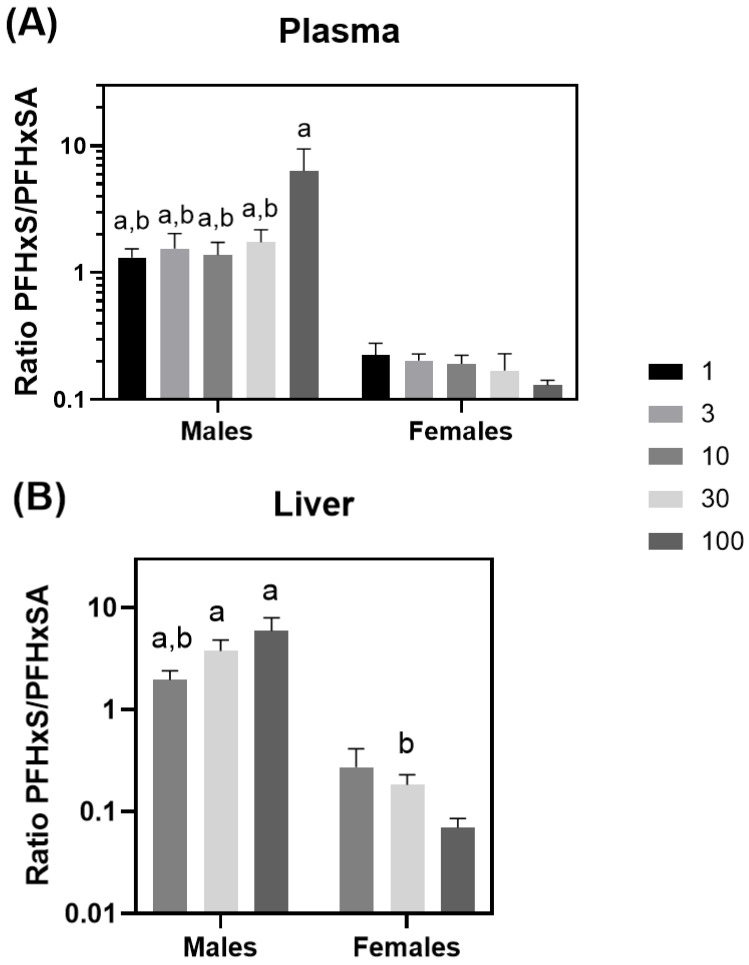
Plasma (**A**) and liver (**B**) PFHxS/PFHxSA ratios. a = statistically different from the same dose in female rats (*p* < 0.05) and b = statistically different from the high dose within the same gender (*p* < 0.05).

Male PFHxS liver concentrations were consistently approximately ten-fold greater than those for females at equivalent dose levels and were statistically different from females at 10, 30, and 100 mg/kg/day (*p* < 0.003) (Table 5). Two-way ANOVA testing indicated that results for both dose and sex were significant (*p* < 0.00044), as was the interaction of the two (*p* < 0.05). Individual rat PFHxS liver data may be found in SI Table S14. Ratios of PFHxS/PFHxSA were significantly different between dose levels (*p* < 0.01) and gender (*p* < 0.0001) for both plasma and liver (Figure 4A,B). Plasma and liver PFHxS/PFHxSA ratios were higher in males than in females (Figure 4A,B). PFHxS concentrations were greater than PFHxSA concentrations in both plasma and liver in males, while the converse was true for females.

Liver-to-plasma partitioning (K_p_) for PFHxSA was assessed for each sex (SI Text S8, Table 6). There were no sex differences in PFHxSA K_p_ values. However, two-way ANOVA testing indicated that dose had a significant impact on the PFHxSA liver-to-plasma partitioning ratio (*p* < 0.0001) and indicated statistically significant interaction between the variables (*p* < 0.005). Refer to SI Table S15 for K_p_ results for individual rats.

### 3.5. In Vitro Toxicokinetics (TK) and In Vitro–In Vivo Extrapolation (IVIVE) of PFHxSA and PFHxS

Plasma protein binding and in vitro hepatocyte clearance experimentally measured for PFHxSA and PFHxS (this study and in [58,59]) were then scaled to estimate hepatic clearance (Cl_hep_; units of L/h), non-metabolic renal clearance, and IVIVE-derived steady state concentrations (C_ss_) in rats and humans [59,62]. In the rat plasma, PFHxS binding was moderately higher (i.e., 25%) than PFHxSA, and non-metabolic renal clearance of both compounds was identical (0.0001 L/h) (Table 7). Hepatic clearance was only observed for PFHxSA in rat hepatocytes. These toxicokinetic characteristics resulted in PFHxS C_ss_ values that are 1.8-fold higher than PFHxSA.

In the human in vitro evaluations, differences in plasma binding drove the differences in C_ss_ values. No hepatic clearance was observed for either compound, whereas a 2.25-fold-higher plasma binding in PFHxSA resulted in a similarly reduced renal clearance. It follows that IVIVE-derived C_ss_ values were also 2.25-fold lower for PFHxS compared to PFHxSA. Assuming a 1 mg/kg/day exposure, C_ss_ values of 1510 and 669 μM were estimated for PFHxSA and PFHxS, respectively. As the IVIVE-derived C_ss_ assumes a 1 mg/kg/day exposure rate, the in vivo-study-derived PFHxSA rat plasma concentrations from Table 2 were considered in Table 7 to evaluate the predictivity of the IVIVE model. The IVIVE-derived C_ss_ predictions for PFHxSA were 6.5–12.1 times higher than the in vivo concentrations for female and male rat plasma, respectively. No such comparison can be made for PFHxS, since those in vivo exposures were secondary to the PFHxSA administration.

## 4. Discussion

Recent in vivo and in vitro studies have attempted to address the dearth of exposure and effects data for emerging PFASs. Non-targeted analysis of PFAS metabolite formation in SD rats following PFHxSA administration revealed substantial conversion of the parent to PFHxS, a persistent sulfonic acid already associated with health effects [45]. Although other PFAS were detected, such as perfluorohexanesulfinic acid and PFHxSA-glucuronide, these comprised less than 3% of the PFAS detected following dosing [45]. Indeed, in the present study, quantification of PFHxS in male rats after 5 days of daily dosing of PFHxSA at 30 and 100 mg/kg/day yielded average plasma concentrations of 136 and 396 μM, respectively. This is contrasted with coincident average PFHxSA plasma levels of 81.2 and 62.1 μM, respectively, where PFHxS plasma concentrations are 1.6- to 6-fold higher. The biotransformation of PFHxSA to the much more stable PFHxS underscores the need to consider how this systemic co-exposure should be interpreted to deconvolute any downstream effects in any similar in vivo PFHxSA studies.

Significant decreases in plasma THs T3 and T4 were observed at the highest dose in both sexes, except for T4 in males, which was decreased starting at 10 mg/kg/day. Reduced rat TH concentrations have been noted previously for PFHxSA [40] and PFHxS [63,64,65], with Auerbach et al. [40] noting a similar T4 response. Apart from the concomitant sex differences regarding higher PFHxS levels and T4 decreases in males and the available literature data, it is difficult to discern whether the T4 effects are due to PFHxSA or PFHxS exposure. Dong et al. [63], in their 28-day, 50 mg/kg/day, PFOS dietary study, noted that male rat TH target genes were not altered in a manner consistent with reduced TH signaling. Rather, genes involved in hepatic metabolism, indicating constitutive androstane receptor/pregnane X-receptor (CAR/PXR) activation and peroxisome proliferator-activated receptor-α (PPAR-α) involvement, suggest the decreases are indirect, secondarily due to enhanced TH metabolic clearance. The derivation of 16.25 mg/kg/day as the benchmark dose for relative liver weight changes following PFHxSA exposure in Auerbach et al. [40] and metabolic enzyme upregulation noted in the underlying transcriptomic data described in Mutlu [55] provides support for a similar mechanism at play in this study. The changes in THs occur at exposures of 10 mg/kg/d or higher in rats. Furthermore, transcriptional pathway changes are observed at doses of less than 1 mg/kg/day in multiple tissues [40,66] suggesting that the TH changes are unlikely to be the critical effect for PFHxSA.

The sex differences noted with regard to PFHxSA and PFHxS dosimetry indicate a likely divergence in metabolism and elimination processes. At doses of 10 mg/kg/day and up, the female rats exhibited, on average, 2.9-fold higher plasma PFHxSA levels than males, in conjunction with an average 5.3-fold lower level of PFHxS (Table 2 and Table 3). These results suggest that males have a heightened capacity to biotransform PFHxSA to PFHxS compared to females. Comparison of PFHxS/PFHxSA concentration ratios in plasma and liver shows a consistent trend across the dose–response, with male ratios approximately 10-fold higher than female ratios in both tissues (Figure 4). A closer look at transcriptomic changes conducted as a part of Mutlu et al. [55] indicate at least a 2-fold increase in mRNAs corresponding to cytochrome P450 (CYP450) hepatic metabolism enzyme isoforms 3A2, 4A1, and 2J4, all with the lowest observed transcriptional effect level of 30 mg/kg/day. These findings, in conjunction with earlier in silico and in vitro findings supporting PFASA metabolism by CYP450s [67,68], provide useful information for future evaluations.

In vivo evaluations of PFAS half-life differences by sex do not reveal consistent trends. The only PFASA study with in vivo data, conducted by Auerbach et al. [40], confirms our observation of longer PFHxSA half-lives in females, in a similar five-day repeat-dose study [55]. Evaluations of PFAS containing sulfonate groups have noted longer half-lives in male rats for PFHxS [69,70,71,72] and perfluorobutane sulfonate [70]. Perfluorohexanoic acid and PFOA exhibited longer half-lives in male SD rats, while PFOS and PFDA half-lives exhibited little difference between sexes [70,73]. Factors that may contribute to these sex differences include hormonal regulation of hepatic metabolism and/or tissue-specific transporters involved in either chemical efflux or reuptake. For example, sexual dimorphism has been noted in the expression and tissue distribution of the cytochrome P450 enzyme family [74], some of which have been noted to metabolize and/or interact with PFAS [75,76,77]. Regarding transporters, earlier toxicokinetic studies revealed that renal clearance was responsible for total clearance, accounting for sex differences for perfluorocarboxylic acids [78]. Moreover, evaluations of rat renal organic anion transporter family revealed these to be under hormonal control, leading to vast sex differences in PFOA half-life [79]. Unfortunately, limited in vivo data exploring these differences for PFAS, in particular the sulfonamides and other emerging PFAS, limit our statements to speculation.

Liver-to-plasma K_p_s indicate PFHxSA partitions more to the blood than liver (Table 6), with K_p_ values ranging from 0.13 to 0.60 in both sexes across all tested doses. Although studies on legacy PFASs, such as PFOA and PFOS, have noted enhanced liver partitioning in male rats, with values ranging from 1.5 to 10.9, a few studies on other PFASs, such as PFBA and PFHxS, indicate decreased liver partitioning, with summary values of 0.16–0.44 across both sexes [80]. Variable partitioning in the literature suggests sex differences might be present for a subset of PFAS, but more studies are needed to confirm [81,82]. Notably, a recent evaluation of human liver/plasma partitioning in bariatric surgery patients showed PFHxS and PFOA K_p_ values of 0.12 and 0.26, respectively, with others (including PFOS, perfluoroheptanoic acid, perfluorodecanoic acid, and perfluoroundecanoic acid) ranging from 1.37 to 3.64 [83]. Ultimately, more data are needed across a range of PFAS to clarify when preferential liver partitioning is occurring, and when sex or species differences may be important in dosimetry data interpretation and extrapolation to inform human risk partitioning.

Comparisons of the in vivo plasma concentrations measured in this study against IVIVE-derived plasma C_ss_ concentrations may provide an indication of a PFAS TK model performance, although with some important caveats. The IVIVE model employs metrics of in vitro hepatic and non-metabolic clearance and plasma protein binding, with other parameters often set to conservative assumptions (e.g., 100% intestinal absorption, no extrahepatic metabolism). For PFAS, these assumptions are likely appropriate: in vivo studies evaluating absorption have shown those measured in rats, including PFOA, PFOS, PFHxS, and perfluorobutane sulfonic acid, to be readily absorbed [81,84,85], as expected given their amphiphilic nature. For stable compounds exhibiting low or no hepatic elimination, extrahepatic metabolism would be negligibly impactful at best. The IVIVE-derived PFHxSA C_ss_ estimates, when compared to the plasma concentrations resulting at the 1 mg/kg/day dose rate, were 6.5–12-fold higher than the measured values. Although an over-prediction, erring on the side of health-protective exposure predictions, this performance is consistent with that observed for non-PFAS chemicals, for which in vivo C_ss_ data were available for comparison [61,86]. It is worth noting that a steady state will not likely be achieved for PFHxSA after just 5 days of dosing, given recent evaluations of in vivo PFAS toxicokinetic data [86,87]. Further, the metabolic enzyme induction that appears to be happening with repeated PFHxSA treatment is another condition that is not incorporated into the IVIVE approach. Enhanced metabolism secondary to enzyme induction would result in a decrease in C_ss_ values. Renal transporter involvement is also not yet considered. Despite these sources of uncertainty, the cross-species C_ss_ comparisons presented here for PFHxSA and PFHxS, where humans are predicted to exhibit 3- to 10-fold higher concentrations than rats, are consistent with reported species differences in other in vivo toxicokinetic analyses [80,88,89]. The ongoing development of more sophisticated cellular or organotypic models to characterize hepatic and renal elimination pathways is poised to improve TK inputs for IVIVE modeling [90,91,92,93]. Refinement of such inputs, across species, will be critical to advancing extrapolations to inform PFAS risk-based decision-making.

PFHxSA biotransformation to PFHxS and the subsequent systematic co-exposures have ramifications for the interpretation of both in vivo and in vitro toxicity evaluations. In in vivo scenarios, the ability to discern the ultimate toxicant initiating a particular perturbation is hampered without simultaneous monitoring of dosimetry of all relevant parent compounds and putative BPs within toxicity evaluations. As real-world exposures always constitute co-exposures to multiple compounds, there is an important opportunity to leverage dosimetric evaluations to better characterize synergistic or additive effects that may be occurring not just following external co-exposures, but also due to internal co-exposures brought on by biotransformation. Beyond PFHxSA, other PFASAs have either been shown to are expected to be hydrolyzed to their respective sulfonic acid metabolites, including PFOSA and N-Ethylperfluorooctane sulfonamide, both of which biotransform to PFOS [67,94]. Also, in vitro and in vivo studies have confirmed the formation of PFOA following metabolism of 8:2 fluorotelomer alcohol [76,95,96]. Tools such as the Chemical Transformation Simulator (CTS: https://qed.epa.gov/cts/; accessed on 11 November 2025) have developed reaction libraries based on peer-reviewed literature reports [97], with a recent update designed to specifically capture PFAS biotransformation pathways [98]. Tools such as these will provide critical information to assist in study design.

In vivo evidence of biotransformation needs to also inform the interpretation of New Approach Method (NAM) data streams, particularly for PFAS, where NAMs are being increasingly employed to address serious data gaps across the larger PFAS universe of thousands of entities [99,100,101]. In most NAMs, in vitro points of departure are derived in systems lacking metabolic capability, so only the toxicity of the parent compound is captured. If the metabolite is the putative toxic agent, or if synergistic effects are initiated with parent-metabolite co-exposures, the in vitro pathways identified and/or the potency metrics derived will be a misrepresentation, both qualitatively and quantitatively, of the in vivo perturbations. Fortunately, strategies to incorporate metabolism within NAM data streams are evolving [102,103]. Alternately, a tiered approach that considers the likelihood of formation of a bioactive metabolite using predictive tools can be incorporated to aid in flagging chemicals requiring further investigation.

## 5. Conclusions

This evaluation extends findings from an earlier non-targeted evaluation wherein in vivo concentrations of PFHxSA and metabolite PFHxS predominated following PFHxSA exposure in an SD rat model. Quantitative measures of these two PFAS confirmed average plasma PFHxS levels ranging from 76.9 to 396 μM following five days of dosing at 10–100 mg/kg/day of PFHxSA in males, underscoring the critical need to consider PFAS co-exposure for this and likely other PFASAs. Furthermore, the pronounced sex differences in PFHxSA/PFHxS dosimetry noted here, and for several PFAS in other studies with only minimal mechanistic evaluation of etiology, indicate a significant data gap requiring further study, as does the relevance of such differences to human evaluations. Ultimately, further study of the kinetic characteristics of biotransformation reactions, as well as the toxicokinetic and toxicodynamic characteristics of the PFAS parent and BPs, should foster a better understanding of their net toxicological effect following real-world exposures.

## Figures and Tables

**Table 1:** Plasma thyroid hormone concentrations following 5-day daily PFHxSA exposure.

Dose Level (mg/kg/day)	Male T3 Conc. (ng/mL)	Female T3 Conc. (ng/mL)	Male T4 Conc. (ng/mL)	Female T4 Conc. (ng/mL)
0	0.815 ± 0.12	0.882 ± 0.23	42.2 ± 4.8	30.1 ± 6.5
0.01	0.733 ± 0.15	0.851 ± 0.14	34.2 ± 3.4	29.8 ± 8.4
0.1	0.676 ± 0.30	0.906 ± 0.16	38.8 ± 17	30.5 ± 5.2
0.3	0.743 ± 0.16	0.931 ± 0.11	37.9 ± 5.9	32.6 ± 4.1
1	0.789 ± 0.21	0.789 ± 0.11	39.3 ± 5.7	33.9 ± 6.2
3	0.754 ± 0.085	0.712 ± 0.13	32.7 ± 7.7	26.8 ± 3.9
10	0.757 ± 0.14	0.759 ± 0.11	24.1 ± 6.7 *	26.8 ± 5.8
30	0.531 ± 0.094	0.697 ± 0.16	14.5 ± 4.6 *	21.9 ± 7.4
100	0.463 ± 0.20 *	0.475 ± 0.18 *	7.6 ± 3.0 *	11.7 ± 4.8 *

Mean ± standard deviation (SD); *n* = 5 per sex per dose level, except *n* = 8 per sex for 0 mg/kg/day. Conc.: Concentration; * Statistically significant compared to controls (*p* < 0.05) by Dunnett’s test. Please see the text for more statistical evaluation details.

**Table 2:** Male and female PFHxSA plasma concentrations following 5-day daily exposure.

Dose Level (mg/kg/day)	Male Plasma Conc. (µM)	Female Plasma Conc. (µM)
0	ND	ND
0.01	0.102 ^†^	0.117 ± 0.043 ^‡^
0.1	0.796 ± 0.28	1.10 ± 0.11
0.3	4.26 ± 0.48	4.34 ± 1.6
1	11.4 ± 2.2	21.3 ± 9.1
3	27.1 ± 8.0	37.1 ± 13
10	56.8 ± 14	64.9 ± 17
30	81.2 ± 20 *	179 ± 66 *

Mean ± SD; *n* = 5 per sex per dose level, except *n* = 8 per sex for the 0 mg/kg/day dose. ND: Not detected above the LOQ; Conc.: Concentration. ^†^ The data are from a single rat with plasma concentrations above the LOQ. ^‡^ The concentration for one sample is estimated based on half the LOQ, as all other samples within the dose level had concentrations above the LOQ. * Statistically significant difference between sexes within the same dose (*p* < 0.05).

**Table 3:** PFHxS plasma concentrations following 5-day daily PFHxSA exposure.

Dose Level (mg/kg/day)	Male Plasma Conc. (µM)	Female Plasma Conc. (µM)
0	ND	ND
0.01	ND	ND
0.1	1.42 ^†^	ND
0.3	7.47 ± 5.3	1.48 ^†^
1	14.7 ± 1.8	4.42 ± 1.2 *
3	40.2 ± 7.8	7.27 ± 1.9 *
10	76.9 ± 20	12.2 ± 2.1 *
30	136 ± 23	29.2 ± 8.7 *
100	396 ± 200 *	30.9 ± 7.7 *

Mean ± SD; *n* = 5 per sex per dose level, except *n* = 8 per sex for the 0 mg/kg/day dose. ND: Not detected above the LOQ; Conc.: Concentration. ^†^ The data are from a single rat with plasma concentrations above the LOQ. * Statistically significant difference between sexes within the same dose (*p* < 0.01).

**Table 4:** PFHxSA liver concentrations following 5-day daily PFHxSA exposure.

Dose Level (mg/kg/day)	Male Liver Conc. (µM)	Female Liver Conc. (µM)
0	ND	ND
0.01	ND	ND
0.1	0.466± 0.19 ^‡^	0.460 ± 0.10
0.3	1.06 ± 0.28	1.69 ± 0.32
1	2.48 ± 0.39	4.20 ± 1.6
3	4.67 ± 0.47	7.91 ± 2.4
10	10.6 ± 2.5	8.41 ± 1.1
30	11.6 ± 2.6	26.1 ± 7.9 *
100	25.7 ± 16	54.0 ± 24

Mean ± SD; *n* = 5 per sex per dose level, except *n* = 8 per sex for the 0 mg/kg/day dose. ND: Not detected above the LOQ; Conc.: Concentration. ^‡^ The concentration for one sample is estimated based on half the LOQ, as all other samples within the dose level had concentrations above the LOQ. * Statistically significant difference between sexes within the same dose (*p* < 0.033).

**Table 5:** PFHxS liver concentrations following 5-day daily PFHxSA exposure.

Dose Level (mg/kg/day)	Male Liver Conc. (µM)	Female Liver Conc. (µM)
0	ND	ND
0.01	ND	ND
0.1	ND	ND
0.3	2.71 ± 2.1 ^‡^	ND
1	5.70 ± 1.4	ND
3	10.8 ± 2.9	ND
10	20.5 ± 3.1 *	2.04 ± 0.89 *^,‡^
30	42.7 ± 9.9 *	4.64 ± 1.5 *
100	117 ± 82 *	4.88 ± 3.3 *

Mean ± SD; *n* = 5 per sex per dose level, except *n* = 8 per sex for the 0 mg/kg/day dose. ND: Not detected above the LOQ; Conc.: Concentration. ^‡^ The concentration for one sample is estimated based on half the LOQ, as all other samples within the dose level had concentrations above the LOQ. * Statistically significant difference between sexes within the same dose (*p* < 0.003).

**Table 6:** PFHxSA liver-to-plasma K_p_ values.

Dose Level (mg/kg/day)	K_p_, Male Rats	K_p_, Female Rats
0.1	0.604 ± 0.22	0.420 ± 0.090
0.3	0.256 ± 0.088	0.431 ± 0.16
1	0.223 ± 0.053	0.210 ± 0.074
3	0.186 ± 0.058	0.222 ± 0.048
10	0.189 ± 0.041	0.135 ± 0.030
30	0.144 ± 0.0075	0.148 ± 0.011
100	0.403 ± 0.23	0.222 ± 0.072

Mean ± SD; *n* = 5 per sex per dose level.

**Table 7:** Toxicokinetic parameters for PFHxSA and PFHxS in rat and human model.

	Rat		Human	
Chemical	PFHxSA	PFHxS	PFHxSA	PFHxS
f_up_	0.0010	0.0008	0.0004 ^1^	0.0009 ^1^
f_ub_	0.0018	0.0014	0.0007 ^1^	0.0016 ^1^
Cl_renal_ (L/h)	0.0001	0.0001	0.0049	0.0110
Cl_hep_ (L/h)	0.0000449	0	0 ^2^	0 ^2^
C_ss_ (µM)	138	243	1510	669
C_plas, female (male)_ (µM)	21.3 ± 9.1 (11.4 ± 2.2)		N/A	N/A

Abbreviations: f_up_: fraction unbound in plasma; f_ub_: fraction unbound in blood; Cl_renal_: renal clearance; Cl_hep_: hepatic clearance; C_ss_: steady-state concentration; C_plas_: Plasma concentration at 1 mg/kg/day in this study (mean ± SD); N/A: Not applicable. Additional details are provided in SI Texts S6 and S7. ^1^ Data from Smeltz et al. [60]; ^2^ data from Crizer et al. [59].

## Data Availability

Considering the funding of this effort by the USEPA and in compliance with the USEPA Public Access policy, the accepted, non-formatted version of the accepted manuscript and any associated data files will be made available on PubMed Central one year after acceptance by the journal.

## Supplementary Materials

The supporting information can be downloaded at https://www.mdpi.com/article/10.3390/toxics13121022/s1. Table S1: Chemical Standards; Table S2: Thyroid hormone chromatography gradient used for plasma analysis; Table S3: Thyroid hormone instrument parameters; Table S4: Thyroid hormone MRM transitions; Table S5: PFHxSA and PFHxS instrument conditions for plasma analysis; Table S6: PFHxSA and PFHxS MRM transitions; Table S7: PFHxS chromatography gradient; Table S8: Dosing solution concentrations of PFHxSA; Table S9: PFHxSA in corn oil stability assessment results; Table S10: Individual rat plasma T3, rT3, andT4 concentrations; Table S11: Individual rat plasma PFHxSA concentrations; Table S12: Individual rat plasma PFHxS concentrations; Table S13: Individual rat liver (wet weight; ww) PFHxSA concentrations; Table S14: Individual rat liver (wet weight) PFHxS concentrations; Table S15: Individual rat liver-to-plasma partitioning coefficients (Kp).
