# Supplementary material for: Evaluations of Sex Differences in Dosimetry in Rats Following Perfluorohexanesulfonamide (PFHxSA) Oral Exposure
Source: Toxics. 2025 Nov 26;13(12):1022. doi: 10.3390/toxics13121022 (PMC12737022; doi:10.3390/toxics13121022)
Supplement: Supplementary file 1 [file toxics-13-01022-s001.zip › toxics-3934409-supplementary.pdf]

Evaluations of Thyroid Effects, Dosimetry, and Toxicokinetics of Perfluorohexanesulfonamide (PFHxSA) Exposure

Supporting Information

Jackson G. Bounds[1], Aero Renyer[2], Jermaine L. Ford,[3] Krishna Ravindra,[1] Michael J. Devito[3], Michael F. Hughes[3], Leah C. Wehmas[3], Amanda A. Brennan[3*], Barbara A. Wetmore[3], and Denise K. MacMillan[3*]

1 Oak Ridge Associated Universities (ORAU), Oak Ridge, TN 37830, USA
2 Oak Ridge Institute for Science and Education (ORISE), Oak Ridge, TN 37830, USA
3 Center for Computational Toxicology and Exposure, Office of Research and Development, U.S. Environmental Protection Agency (USEPA), Durham, NC 27709, USA
* Correspondence: denisekmacmillan@gmail.com ; Tel.: +1-919-541-4128; brennan.amanda@epa.gov ; Tel.: 1-919-541-2323

Table of Contents

Supplementary Texts:

Text S1 Solvents and Additives for Analytical Chemistry

Text S2 Dosing Solution Determination and Stability Assessment

Text S3 Plasma and Liver Dosimetry

Text S4 Thyroid Hormone Analysis

Text S5 In vivo statistics

Text S6 Hepatocyte Metabolic Stability Assay Materials, Chemicals, and Calculations

Text S7 In Vitro–In Vivo Extrapolation (IVIVE) Calculations

Text S8 Liver-to-Plasma Partitioning Coefficient Calculation

Supplementary Tables:

Table S1 Chemical Standards

Table S2 Thyroid hormone chromatography gradient used for plasma analysis.

Table S3 Thyroid hormone instrument parameters

Table S4 Thyroid hormone MRM transitions

Table S5 PFHxSA and PFHxS instrument conditions for plasma analysis

Table S6 PFHxSA and PFHxS MRM transitions

Table S7 PFHxS chromatography gradient

Table S8 Dosing solution concentrations of PFHxSA

Table S9 PFHxSA in corn oil stability assessment results

Table S10 Individual rat plasma T3, rT3, andT4 concentrations

Table S11 Individual rat plasma PFHxSA concentrations.

Supporting Information

Table S12 Individual rat plasma PFHxS concentrations

Table S13 Individual rat liver (wet weight; ww) PFHxSA concentrations

Table S14 Individual rat liver (wet weight) PFHxS concentrations

Table S15 Individual rat liver-to-plasma partitioning coefficients (Kp)

Supporting Texts

Text S1 Solvents and Additives for Analytical Chemistry

Solvents used for sample preparation and mobile phases include methanol (MeOH), water (H_2O), and acetonitrile (ACN), which were LC/MS-grade and purchased from Honeywell Burdick & Jackson (Charlotte, NC, USA). Ammonium formate (BioUltra purity) and ammonium acetate (99% pure) were used as mobile phase additives and obtained from Millipore Sigma (St. Louis, MO, USA). Formic acid (FA; 97.5% pure) was also used as an additive; it was purchased from Honeywell Fluka. Detailed information for chemicals used for each analysis may be found in Table S1.

Text S2 Dosing-Solution Determination and Stability Assessment

Dosing solutions were extracted by liquid–liquid extraction using 50 uL aliquots of the dosing solutions and 950 uL of 0.1M formic acid in acetonitrile, vortexing the mixture at 2000 rpm, and centrifuging at 5400 × g for 10 minutes. The supernatants were drawn off and added to Waters Oasis Prime HLB 3cc 60mg (Waters, Milford, MA, USA) solid phase extraction (SPE) cartridges. Supernatants were allowed to elute through the cartridge via gravity. Finally, 100 uL of eluent was diluted using 100 uL of 1% formic acid in water. Extracts were further diluted with the extracted method blank (corn oil) to bring the concentrations to within the calibration range. Sample extracts were analyzed using the same LC/MS/MS method outlined for PFHxSA plasma and liver in Text S4. Samples were quantitated against a matrix-matched calibration curve containing a minimum of 5 points spanning 50-500 ng/mL in extract. Calibration curves had a quadratic fit with a correlation coefficient ≥ 0.995. A continuing calibration verification (CCV) sample and laboratory control sample (LCS) were injected at the start and end of each analytical batch to confirm the validity of the calibration curve and sample quantitation. Acceptance criteria for the CCV and LCS were readback concentrations ± 30% of nominal concentration. The method blank acceptance criterion was a readback concentration of PFHxSA < 0.5x the limit of quantitation (LOQ) of the method. The LOQ was 50 ng/mL in the extract. Samples were injected in sequential order (e.g., r01, r02, r03, …, etc.); no duplicate injections of samples were performed.

Assessment of the stability of PFHxSA in corn oil was conducted using the same extraction method as for dosing solutions. Analysis of PFHxSA in corn oil was conducted using the same LC/MS/MS conditions as PFHxSA dosimetry in plasma and liver (Text S4). The average ratio of PFHxSA peak area to PFOSA internal standard peak area for three replicate injections was divided by the average ratio for Day 0 to derive the percentage of recovery for a given day. No calibration curve was generated for the Stability Assessment. For quality control, the extraction blank needed to have a PFHxSA signal that was ≤ 10% the peak area of PFHxSA on Day 0; the daily LCS was acceptable with a PFHxSA/PFOSA area ratio within 30% of the Day 0 LCS.

Text S3 Plasma and Liver Dosimetry

Sprague Dawley rat plasma (pooled, mixed sex) collected with ethylenediaminetetraacetic acid (EDTA) and sterile filtered (0.2 μm) and Sprague Dawley rodent liver (mixed sex) were obtained from BioIVT (Westbury, NY, USA) for use as the control matrix for generating calibration curves and as the matrix blank.

Plasma sample aliquots (25 μL) were spiked with the internal standard PFOSA, as an isotopically labeled PFHxSA standard was not available. Aliquots were denatured with 105 μL of 0.1 M FA in ACN. Samples were vortexed, stored at -20 °C for 30 min, then centrifuged at 10,000 × g for 30 min. Supernatants were collected and stored at -20 °C prior to analysis. Supernatants were diluted 1:1 with 1% FA in water immediately prior to analysis.

Liver sample aliquots (~10 mg) were placed into Eppendorf Safe-Lock 2 mL tubes (Hamburg, DE), spiked with PFOSA, and then diluted with 1005 µL of 0.1 M FA in ACN and stored at -80 °C for 10 min. Samples were homogenized using an OMNI Bead Ruptor 24 (OMNI International, Kennesaw, GA, USA) with the following settings: 2 cycles, 60-second cycle time, 5.5 m/sec, and a 30-second dwell time. They were centrifuged at 26,000 × g for 10 min. Supernatants were collected and stored at -20 °C prior to analysis. Supernatants were diluted 1:1 with 1% FA in water immediately prior to analysis.

Samples were quantitated against matrix-matched calibration curves containing a minimum of 5 points spanning the range 243-2499 ng/mL in plasma and 1.4-36.8 ng/mL in extract in liver. Calibrants were prepared by spiking commercial rat plasma or liver with PFHxSA and PFOSA and then extracted in the same manner as study samples. Calibration curves had a quadratic fit with a correlation coefficient ≥ 0.995. An ICV sample was prepared from a second source standard and analyzed with the calibration curve to confirm its validity. Acceptance criterion for the ICV was a recovery of ± 20% of the nominal concentration of the ICV as quantitated from the calibration curve. Method blank, CCV, and LCS were injected at the beginning of every extraction batch and reinjected after every 30 samples to confirm the validity of the calibration curve and sample quantitation. If fewer than 30 samples were injected, all samples were bracketed by injections of the CCV at the beginning and end of the analysis batch. Acceptance criteria for the CCV and LCS were ± 30% of nominal concentration. Method blanks were acceptable with PFHxSA signal < 0.5x the LOQ. Samples were injected in order of increasing dose. Quantitation of PFHxS in liver and plasma was performed using the same QC criteria and extraction methods as PFHxSA, only the internal standard was supplied in the form of a Wellington Laboratories isotopically labeled MPFAC-24ES PFAS mixture containing $^{13}C_3$-PFHxS. Samples were quantitated against PFHxS matrix-matched calibration curves containing a minimum of 5 points spanning the range 544-60354 ng/mL in plasma and 6.93-754.4 ng/mL in extract in liver. An ICV sample was prepared from a second source standard and analyzed with the calibration curves to confirm their validity. The acceptance criterion for the ICV was a recovery of ± 20% of the nominal concentration of the ICV as quantitated from the calibration curve.

Text S4 Thyroid Hormone Analysis

A 20 µL aliquot of plasma from each sample was loaded into individual wells of a 96-well collection plate, spiked with 5 µL of a 40 ng/mL $^{13}C_6$-T3, $^{13}C_6$-rT3 and $^{13}C_6$-T4 mixed solution followed by 1N hydrochloric acid (HCl) (20 µL) (36.5-38%, Fisher Scientific. Waltham, MA, USA), H_2O (100 µL), and a 50:50 H_2O/ACN solution (vol/vol) (60 µL) containing 0.1% formic acid (FA). Samples were vortexed, incubated at 37 °C for 2 hours, and brought to room temperature. Samples were diluted with an aqueous 0.1% acetic acid solution (LC/MS grade, Honeywell Fluka, Charlotte, NC, USA) and vortexed. SPE well plates (Evolute CX, 96-well SPE plate, 10 mg, 1 mL, Biotage, Charlotte, NC, USA), processed with a positive pressure manifold, were conditioned with methanol followed by an aqueous 0.1% acetic acid solution prior to sample loading with low pressure. Plate wells were washed with 0.1% acetic acid, followed by methanol. Thyroid hormones were eluted into a collection plate with 2.5% ammonium hydroxide (NH_4OH) (28-30%, Thermo Fisher Scientific, Waltham, MA, USA) in methanol. Extracts were then evaporated to dryness with nitrogen using a Turbovap (Biotage), then reconstituted in 100 µL of 25:75 ACN/H_2O (vol/vol) with 0.1% acetic acid. Extracts were stored in amber micro-sampling vials (Agilent Technologies, Santa Clara, CA, USA) prior to instrumental analysis.

Samples were quantitated against matrix-matched calibration curves containing a minimum of 5 points spanning the range 0.005 – 25.00 ng/mL. Calibration standards were made by combining T3, rT3, and T4 analytes with the $^{13}C_6$-T3, $^{13}C_6$-rT3, and $^{13}C_6$-T4 internal standards, then with commercial rat plasma. The standards were extracted using the above procedure. Calibration curves had a quadratic fit

with a correlation coefficient ≥ 0.995. An independent calibration verification (ICV) sample was prepared from a second source standard and analyzed with the calibration curve to confirm its validity. The acceptance criterion for the ICV was a recovery of ± 20% of the nominal concentration of the ICV as quantitated from the calibration curve. Matrix blanks were analyzed with each sample set and were below the limit of quantitation, 0.04 ng/mL plasma. The matrix blank was analyzed at the beginning of the analytical run. A CCV and LCS were injected at the start of each analytical batch and were reinjected every 24 samples to confirm the validity of the calibration curve and sample quantitation. Acceptance criteria for the CCV and LCS were readback concentrations ± 30% of nominal concentration. The method blank acceptance criterion was a readback concentration of each thyroid hormone <0.5x the LOQ of the method. Samples were injected in sequential order (e.g., r01, r02, r03, etc.); no duplicate injections of samples were performed.

Text S5 In vivo statistics

In vivo results are reported as the mean ± one standard deviation (SD) of biological replicate data. Data were further analyzed via two-way analysis of variance (ANOVA) and Dunnett's Test using an α = 0.05 significance level in GraphPad Prism v9.5.1.

The percentage dose of PFHxSA in plasma was calculated using the observed plasma concentrations and the average plasma volume for Sprague Dawley rats. The density of plasma was then used to convert from mL plasma to grams plasma for ease of comparison to the liver percent dose data. The average percent dose of PFHxSA in the liver was calculated using the observed liver concentrations and liver weights. These calculations allow for a comparison of the potential saturation of available proteins in
both matrices.

Text S6 Hepatocyte Metabolic Stability Assay Materials, Chemicals, and Calculations

Pooled human and rat cryopreserved primary hepatocyte suspensions were both obtained from BioIVT, a US Food and Drug Administration-licensed and inspected donor center, and produced using non-transplantable tissue. The human 50-donor pool selected from BioIVT's commercially available, pre-pooled lots was confirmed to have 85% post-thaw viability on the day of the experiment using trypan-blue exclusion. The rodent suspension comprised a 24-donor pool of mixed sex Sprague Dawley rat hepatocytes and was confirmed to have 78% viability on the day of the experiment. Vendor-generated metabolic characterization information was reviewed for both lots and deemed acceptable prior to study start. William's E media, dexamethasone (98% pure), and cell maintenance cocktail B were obtained from Thermo Fisher Scientific (Waltham, MA, USA), and the OptiThaw hepatocyte kit from Sekisui/Xenotech (Tokyo, Japan) and the trypan blue solution from Bio-Rad (Hercules, CA, USA). PFHxSA and PFHxS stocks used for these assays were procured through US EPA contract #68HE0D18D0001 with Evotec Inc. (Branford, CT, USA), which provided dosing solutions solubilized in dimethyl sulfoxide (DMSO). Mass-labeled ^{13}C3-PFHxS (> 98% pure) and $^{13}C_8$-PFOSA (> 98% pure), provided within a standard solution mixture (MPFAC-24ES) obtained from Wellington Laboratories, served as internal standards. Propanolol (≥ 98% pure) from Millipore Sigma and phenacetin (≥ 98% pure) from Sigma Aldrich were used as assay reference compounds. Hepatic metabolic clearance data were plotted in semi-log format (ln concentration vs. time) with three replicates at each time point as previously described [61]. Linear regression analysis in conjunction with a standard F-test was used to determine whether the slope of the line (indicative of chemical clearance) was significantly different from 0. Equations (A and B) described below were used to calculate chemical half-life ($T_{½}$) and intrinsic clearance (Cl_{int}) with units of μL/ (minute*million

hepatocytes). In equation (C), the scalar 2000 is used to adjust the assay cell number to be consistent with units of 1 million cells in the Cl_{int} equation.

$$(\mathbf{A})\ k = -(slope)$$

$$(\mathbf{B})\ T_{1/2} = \frac{0.693}{k}$$

$$(\mathbf{C})\ Cl_{int} = \frac{(2000 * 0.693)}{T_{1/2}}$$

Text S7 In Vitro-In Vivo Extrapolation (IVIVE) Calculations

The pharmacokinetic equation used to estimate expected steady-state concentrations (C_{ss}) is based on zero-order uptake of a daily dose from the gut (assuming 100% bioavailability) with both nonmetabolic renal clearance (Cl_{renal}) and hepatic clearance ($Cl_{hepatic}$) (Equation D) [61]. The chemical input rate (k_o) is the product of the intake dosage and the model body weight; this represents the numerator of Equation D. The Cl_{renal} calculation is shown in the first part of the denominator of Equation D; it is the product of the species-dependent glomerular filtration rate (GFR) and the unbound fraction in blood (f_{ub}) of the parent compound. The $Cl_{hepatic}$ calculation is shown in the second part of the denominator of Equation D; it is the product of the species-dependent liver blood flow constant, the f_{ub} of the parent compound, and the experimentally derived intrinsic clearance (shown in Equation C) divided by the sum of these three values. All values used in Equation D are for first-order conditions of metabolism in the liver.

$$(\mathbf{D})\ C_{ss} = \frac{k_0}{GFR * f_{ub} + \frac{Q1 * f_{ub} * Cl_{int}}{Q1 + f_{ub} + Cl_{int}}}$$

Text S8 Liver-to-Plasma Partitioning Coefficient Calculation

Concentrations (μM) of PFHxSA in liver and plasma were used to calculate experimental liver-to-plasma (K_p) partitioning values for both sexes across all dose levels using Equation E.

$$(\mathbf{E})\ \mathrm{K}_p = \frac{Concentration_{organ}}{Concentration_{plasma}}$$

Supplementary Tables

Table S1. Chemical Standards

Chemical Name	CASRN	DTXSID	Vendor	Purity	Experiment
Perfluorohexanesulfonamide (PFHxSA)	41997-13-1	50469320	Synquest Laboratories	99%	Internal dose and Dosing solution confirmation
Perfluorooctanesulfonamide (PFOSA)	754-91-6	3038939	Synquest Laboratories	87%	Internal dose
Perfluorohexanesulfonate (PFHxS)	108427-53-8	80873012	Synquest Laboratories	95%	Internal dose
$^{13}C_3$ Sodium perfluorohexane sulfonate ($^{13}C_3$-PFHxS)	2708218-86-2	901337562	Wellington Laboratories	> 98%	Internal dose
3,3′,5-Triiodothyronine (T3)	6893-02-3	8023216	Cerilliant Corporation	> 99%	Thyroid Hormone
$^{13}C_6$-T3	1217473-60-3	60746331	Cerilliant Corporation	> 98%	Thyroid Hormone
3,3′,5′-Triiodothyronine (rT3)	5817-39-0	3046908	Cerilliant Corporation	> 98%	Thyroid Hormone
$^{13}C_6$-rT3	1217676-14-6	20746332	Cerilliant Corporation	> 99%	Thyroid Hormone
L-thyroxine (T4)	300-30-1	0023662	Cerilliant Corporation	> 98%	Thyroid Hormone
$^{13}C_6$-T4	N/A	N/A	Cerilliant Corporation	> 98%	Thyroid Hormone

Table S2. Thyroid hormone chromatography gradient used for plasma analysis

Time (min)	%A (H_2O + 0.1% formic acid)	%B (MeOH + 0.1% formic acid)
0.01	70	30
3.89	30	70
4.66	30	70
4.67	10	90
6.22	10	90
6.23	70	30
8.55	70	30
8.56	System Controller stop	

MeOH: Methanol

Table S3. Thyroid hormone instrument parameters

Sciex 6500+ Parameter	Setting
Source	ESI
Polarity	Positive
Scan Type	MRM
Source Temperature (°C)	500
Spray Voltage (kV)	5.5
Curtain Gas (psi)	35
Ion Source Gas 1 (psi)	90
Ion Source Gas 2 (psi)	80
Collision Gas	Medium
Detection Window (sec)	80
Scan Time (sec)	0.33

Table S4. Thyroid hormone MRM transitions

Analyte	Precursor Ion	Fragment Ion	Declustering Potential (V)	Collision Energy (CE)	Transition Type
T3	651.80	605.7	100	100.0	Quant
	651.80	478.9	100	50.5	Qual
^{13}C-T3	657.80	605.7	100	33.0	IS
rT3	651.75	605.7	100	34.0	Quant
	651.75	508.1	100	35.0	Qual
^{13}C-rT3	657.80	605.7	100	33.0	IS
T4	777.70	731.7	100	40.0	Quant
	777.70	605.1	100	58.3	Qual
^{13}C-T4	783.70	737.7	100	53.0	IS

MRM: Multiple Reaction Monitoring; **Quant:** Quantitation transition; **Qual:** Confirmation transition; **IS:** Internal standard transition

Table S5. PFHxSA and PFHxS instrument conditions for plasma analysis

Sciex 4000 Parameter	Setting
Source	ESI
Polarity	Negative
Scan Type	MRM
Source Temperature (°C)	400
Spray Voltage (kV)	-3.5
Curtain Gas (psi)	45
Ion Source Gas 1 (psi)	45
Ion Source Gas 2 (psi)	45
Collision Gas	Medium

Table S6. PFHxSA and PFHxS MRM transitions

Analyte	Precursor Ion	Fragment Ion	Declustering Potential (V)	Collision Energy (CE)	Transition Type
PFHxSA	398.0	78.0	-90	-65	Quant
	398.0	64.0	-90	-125	Qual
PFOSA	498.0	78.0	-110	-78	IS
PFHxS	399.0	80.0	-90	-75	Quant
	399.0	99.0	-90	-52	Qual
$^{13}C_3$-PFHxS	402.0	80.0	-90	-90	IS

MRM: Multiple Reaction Monitoring; **Quant:** Quantitation transition; **Qual:** Confirmation transition; **IS:** Internal standard transition

Table S7. PFHxS Chromatography Gradient

Time (min)	%A (95:5 H_2O:MeOH + 10 mM Ammonium acetate)	%B (50:50 MeOH:ACN + 10mM Ammonium acetate)
0.01	100	0
1	100	0
3	55	45
10	0	100
13.5	10	90
13.51	100	0
16.5	100	0

MeOH: Methanol; **ACN:** Acetonitrile

Table S8. Dosing solution concentrations of PFHxSA

Nominal conc., μg/mL	Nominal conc., μM	Measured conc., μg/mL	Measured conc., μM	Percent recovery
Control	0	ND	ND	-
2	5.01	2.07	5.18	104
20	50.1	20.7	51.9	104
60	150	59.7	150	99.5
200	501	184	461	92.0
600	1,500	555	1,390	92.5
2,000	5,010	1,920	4,820	96.2
6,000	15,000	8,050	20,170	134
20,000	50,100	18,050	45,230	90.3

Conc.: Concentration

ND: Not detected above the Limit of Quantitation, 0.05 μg/mL = 0.125 μM

Table S9. PFHxSA in Corn Oil Stability Assessment Results

Conc. (μg/mL)	Storage Temperature (°C)	Replicate injection	Replicate percent recoveries: Day 5	Day 7	Day 14	Mean$_{D14}$	%RE	%RSD$_{D14}$
2	4	1	90.8	78.3	77.3	79.3	20.7	4.0
		2	94.3	79.5	77.6			
		3	97.9	79.3	83.0			
	RT	1	97.4	81.3	97.0	97.3	2.7	4.4
		2	96.8	85.5	93.3			
		3	96.1	80.6	102			
20,000	4	1	110	119	130	128	27.8	1.5
		2	119	118	126			
		3	110	116	128			
	RT	1	97.3	90.1	88.0	88.6	11.4	1.9
		2	103	94.3	87.2			
		3	98.1	88.4	90.5			

Conc.: Concentration; **RT:** Room Temperature; **Mean$_{D14}$:** Mean of percent recoveries for Day 14 replicates; **%RSD$_{D14}$:** Percent relative standard deviation for Day 14 replicates; **%RE:** Percent Relative error

Table S10. Individual rat plasma T3, rT3, and T4 concentrations

Dose Level (mg/kg/day)	Male Rat ID	T3 Conc. (ng/mL)	rT3 Conc. (ng/mL)	T4 Conc. (ng/mL)	Female Rat ID	T3 Conc. (ng/mL)	rT3 Conc. (ng/mL)	T4 Conc. (ng/mL)
0	R001	0.886	0.058	44.0	R049	0.517	ND	27.7
0	R002	0.982	ND	47.8	R050	1.11	ND	35.3
0	R003	0.641	ND	42.4	R051	0.769	ND	24.3
0	R004	0.764	ND	33.0	R052	0.590	ND	25.0
0	R005	0.700	ND	40.2	R053	1.04	ND	42.7
0	R006	ND	0.060	N/A	R054	0.907	ND	23.7
0	R007	0.870	0.094	46.3	R055	1.07	ND	30.9
0	R008	0.864	0.047	41.9	R056	1.05	ND	31.5
0.01	R009	0.778	ND	28.9	R057	1.01	ND	34.0
0.01	R010	0.705	ND	33.6	R058	0.848	ND	19.8
0.01	R011	0.796	ND	37.6	R059	0.785	ND	26.5
0.01	R012	0.496	ND	34.2	R060	0.962	ND	41.8
0.01	R013	0.891	ND	36.4	R061	0.657	ND	27.1
0.1	R014	ND	ND	N/A	R062	0.743	ND	27.6
0.1	R015	0.226	ND	14.9	R063	0.859	ND	34.2
0.1	R016	0.817	ND	43.0	R064	1.16	ND	32.6
0.1	R017	0.797	0.050	42.8	R065	0.914	ND	22.9
0.1	R018	0.865	ND	54.6	R066	0.853	ND	35.3
0.3	R019	0.547	ND	37.1	R067	0.803	ND	34.1
0.3	R020	0.754	ND	31.3	R068	1.03	ND	27.8
0.3	R021	0.698	ND	35.7	R069	0.876	ND	37.3
0.3	R022	0.980	ND	38.2	R070	0.881	ND	28.9
0.3	R023	0.738	4.23	47.3	R071	1.06	ND	34.9
1	R024	0.461	0.056	33.1	R072	0.903	ND	30.0
1	R025	0.947	ND	43.1	R073	0.783	ND	35.1
1	R026	0.705	ND	33.9	R074	0.894	ND	44.3
1	R027	0.984	ND	40.2	R075	0.661	ND	30.6
1	R028	0.846	ND	46.1	R076	0.704	ND	29.5
3	R029	0.773	ND	34.2	R077	0.875	ND	24.0
3	R030	0.768	ND	42.9	R078	0.522	ND	28.6
3	R031	0.613	ND	25.6	R079	0.774	ND	25.1
3	R032	0.844	ND	24.5	R080	0.707	ND	32.7
3	R033	0.772	ND	36.4	R081	0.680	ND	23.6
10	R034	0.959	ND	34.9	R082	0.938	ND	34.3
10	R035	0.566	ND	19.7	R083	0.711	ND	19.1
10	R036	0.773	ND	26.2	R084	0.687	ND	29.2
10	R037	0.714	ND	20.5	R085	0.662	ND	23.3
10	R038	0.770	ND	19.1	R086	0.797	ND	28.4
30	R039	0.536	ND	19.6	R087	0.785	ND	16.2
30	R040	0.547	ND	16.9	R088	0.453	ND	12.7
30	R041	0.607	ND	15.6	R089	0.686	ND	22.3
30	R042	0.594	ND	12.8	R090	0.682	ND	28.0
30	R043	0.372	ND	7.6	R091	0.880	ND	30.0
100	R044	0.315	ND	4.6	R092	0.481	ND	10.7
100	R045	0.398	ND	4.9	R093	0.647	ND	17.9
100	R046	0.266	ND	7.1	R094	0.187	ND	6.21
100	R047	0.721	ND	10.3	R095	0.599	ND	15.1
100	R048	0.617	0.058	11.1	R096	0.460	ND	8.35

ND: Analyte was not detected above the Limit of Quantitation (LOQ): 0.04 ng/mL.

Table S11. Individual rat plasma PFHxSA concentrations

Dose Level (mg/kg/day)	Male Rat ID	Male Plasma Conc. (ng/mL)	Male Plasma Conc. (μM)	Female Rat ID	Female Plasma Conc. (ng/mL)	Female Plasma Conc. (μM)
0	R001	ND	ND	R049	ND	ND
0	R002	ND	ND	R050	ND	ND
0	R003	ND	ND	R051	ND	ND
0	R004	ND	ND	R052	ND	ND
0	R005	ND	ND	R053	ND	ND
0	R006	ND	ND	R054	ND	ND
0	R007	ND	ND	R055	ND	ND
0	R008	ND	ND	R056	ND	ND
0.01	R009	ND	ND	R057	20‡	0.0501‡
0.01	R010	40.6	0.1017	R058	47.4	0.119
0.01	R011	ND	ND	R059	43.3	0.109
0.01	R012	ND	ND	R060	56.7	0.142
0.01	R013	ND	ND	R061	65.0	0.163
0.1	R014	288	0.723	R062	410	1.03
0.1	R015	194	0.487	R063	379	0.951
0.1	R016	490	1.23	R064	487	1.22
0.1	R017	346	0.867	R065	460	1.15
0.1	R018	2670	0.676	R066	458	1.15
0.3	R019	1390	3.49	R067	1280	3.21
0.3	R020	1790	4.49	R068	2460	6.17
0.3	R021	1640	4.12	R069	1610	4.04
0.3	R022	1810	4.54	R070	1010	2.53
0.3	R023	1860	4.66	R071	2300	5.76
1	R024	4950	12.4	R072	5900	14.8
1	R025	3630	9.09	R073	4500	11.3
1	R026	5750	14.4	R074	13600	34.1
1	R027	3710	9.31	R075	10400	26.1
1	R028	4750	11.9	R076	8200	20.4
3	R029	9190	23.0	R077	20100	50.4
3	R030	12600	31.5	R078	6900	17.4
3	R031	7930	19.9	R079	13500	33.9
3	R032	15600	39.0	R080	15900	39.9
3	R033	8760	22.0	R081	17600	44.1
10	R034	17700	44.3	R082	19600	49.0
10	R035	22700	56.8	R083	19000	48.2
10	R036	18200	45.6	R084	29000	72.8
10	R037	23300	58.5	R085	26600	66.8
10	R038	31400	78.8	R086	34900	87.5
30	R039	27700	69.4	R087	61800	155
30	R040	36000	90.1	R088	118000	296
30	R041	23700	59.3	R089	54600	137
30	R042	30900	77.4	R090	62200	156
30	R043	43900	110	R091	60600	152
100	R044	29500	74.0	R092	111000	278
100	R045	25200	63.2	R093	79600	199
100	R046	22500	56.4	R094	111000	275
100	R047	21300	53.4	R095	97600	245
100	R048	25300	63.5	R096	75600	189

ND: Analyte not detected above the Limit of Quantitation (LOQ): 0.100 μM.
‡Concentration estimated based on half the LOQ.

Table S12. Individual rat plasma PFHxS concentrations

Dose Level (mg/kg/day)	Male Rat ID	Male Plasma Conc. (ng/mL)	Male Plasma Conc. (µM)	Female Rat ID	Female Plasma Conc. (ng/mL)	Female Plasma Conc. (µM)
0	R001	ND	ND	R049	ND	ND
0	R002	ND	ND	R050	ND	ND
0	R003	ND	ND	R051	ND	ND
0	R004	ND	ND	R052	ND	ND
0	R005	ND	ND	R053	ND	ND
0	R006	ND	ND	R054	ND	ND
0	R007	ND	ND	R055	ND	ND
0	R008	ND	ND	R056	ND	ND
0.01	R009	ND	ND	R057	ND	ND
0.01	R010	ND	ND	R058	ND	ND
0.01	R011	ND	ND	R059	ND	ND
0.01	R012	ND	ND	R060	ND	ND
0.01	R013	ND	ND	R061	ND	ND
0.1	R014	ND	ND	R062	ND	ND
0.1	R015	ND	ND	R063	ND	ND
0.1	R016	568	1.42	R064	ND	ND
0.1	R017	ND	ND	R065	ND	ND
0.1	R018	ND	ND	R066	ND	ND
0.3	R019	6644	16.6	R067	ND	ND
0.3	R020	2890	7.22	R068	ND	ND
0.3	R021	1410	3.52	R069	ND	ND
0.3	R022	2460	6.15	R070	ND	ND
0.3	R023	1534	3.83	R071	591	1.48
1	R024	5462	13.7	R072	1689	4.22
1	R025	4961	12.4	R073	1042	2.61
1	R026	6268	15.7	R074	1845	4.61
1	R027	5986	15.0	R075	2323	5.81
1	R028	6814	17.0	R076	1950	4.87
3	R029	11871	29.7	R077	3359	8.40
3	R030	19232	48.1	R078	1679	4.20
3	R031	18573	46.4	R079	2694	6.73
3	R032	16775	41.9	R080	3129	7.82
3	R033	13886	34.7	R081	3679	9.19
10	R034	26851	67.1	R082	4202	10.5
10	R035	35716	89.3	R083	4411	11.0
10	R036	20989	52.5	R084	4314	10.8
10	R037	41933	105	R085	5395	13.5
10	R038	28301	70.7	R086	6121	15.3
30	R039	54418	136	R087	16957	42.4
30	R040	41006	102	R088	13423	33.5
30	R041	54617	137	R089	8794	22.0
30	R042	54953	137	R090	8866	22.2
30	R043	67025	168	R091	10396	26.0
100	R044	163742	409	R092	15788	39.5
100	R045	300061	750	R093	8668	21.7
100	R046	106239	266	R094	14885	37.2
100	R047	104121	260	R095	12749	31.9
100	R048	118486	296	R096	9817	24.5

ND: Analyte not detected above the Limit of Quantitation (LOQ): 1.38 µM.

Table S13. Individual rat liver (wet weight) PFHxSA concentrations

Dose Level (mg/kg/day)	Male Rat ID	Male Liver Conc. (ng/mg)	Male Liver Conc. (μM)	Female Rat ID	Female Liver Conc. (ng/mg)	Female Liver Conc. (μM)
0	R001	ND	ND	R049	ND	ND
0	R002	ND	ND	R050	ND	ND
0	R003	ND	ND	R051	ND	ND
0	R004	ND	ND	R052	ND	ND
0	R005	ND	ND	R053	ND	ND
0	R006	ND	ND	R054	ND	ND
0	R007	ND	ND	R055	ND	ND
0	R008	ND	ND	R056	ND	ND
0.01	R009	ND	ND	R057	ND	ND
0.01	R010	ND	ND	R058	ND	ND
0.01	R011	ND	ND	R059	ND	ND
0.01	R012	ND	ND	R060	ND	ND
0.01	R013	ND	ND	R061	ND	ND
0.1	R014	0.191	0.479	R062	0.174	0.436
0.1	R015	0.0842‡	0.211‡	R063	0.196	0.491
0.1	R016	0.156	0.391	R064	0.239	0.599
0.1	R017	0.298	0.746	R065	0.135	0.338
0.1	R018	0.201	0.503	R066	0.173	0.434
0.3	R019	0.503	1.26	R067	0.811	2.03
0.3	R020	0.241	0.604	R068	0.587	1.47
0.3	R021	0.517	1.30	R069	0.783	1.96
0.3	R022	0.462	1.16	R070	0.504	1.26
0.3	R023	0.399	1.00	R071	0.693	1.74
1	R024	0.821	2.06	R072	1.16	2.91
1	R025	0.846	2.12	R073	1.01	2.52
1	R026	1.00	2.51	R074	1.66	4.17
1	R027	1.09	2.73	R075	1.90	4.76
1	R028	1.18	2.97	R076	2.65	6.64
3	R029	1.78	4.45	R077	3.17	7.94
3	R030	1.92	4.81	R078	1.94	4.86
3	R031	1.86	4.67	R079	2.55	6.40
3	R032	1.63	4.08	R080	3.81	9.55
3	R033	2.14	5.36	R081	4.30	10.8
10	R034	3.37	8.44	R082	3.58	8.98
10	R035	5.65	14.2	R083	2.78	6.97
10	R036	3.24	8.13	R084	3.45	8.65
10	R037	4.53	11.4	R085	3.02	7.58
10	R038	4.26	10.7	R086	3.93	9.86
30	R039	4.32	10.8	R087	9.54	23.9
30	R040	4.86	12.2	R088	15.8	39.6
30	R041	3.36	8.43	R089	7.72	19.4
30	R042	4.47	11.2	R090	10.1	25.2
30	R043	6.25	15.7	R091	8.88	22.3
100	R044	14.9	37.4	R092	20.7	52.0
100	R045	19.0	47.6	R093	12.3	30.8
100	R046	6.87	17.2	R094	37.7	94.6
100	R047	4.89	12.3	R095	20.9	52.3
100	R048	5.61	14.1	R096	16.1	40.3

ND: Analyte not detected above the Limit of Quantitation (LOQ): 0.351 μM
‡Concentration estimated based on half the LOQ.

Table S14. Individual rat liver (wet weight) PFHxS concentrations

Dose Level (mg/kg/day)	Male Rat ID	Male Liver Conc. (ng/mg)	Male Liver Conc. (μM)	Female Rat ID	Female Liver Conc. (ng/mg)	Female Liver Conc. (μM)
0	R001	ND	ND	R049	ND	ND
0	R002	ND	ND	R050	ND	ND
0	R003	ND	ND	R051	ND	ND
0	R004	ND	ND	R052	ND	ND
0	R005	ND	ND	R053	ND	ND
0	R006	ND	ND	R054	ND	ND
0	R007	ND	ND	R055	ND	ND
0	R008	ND	ND	R056	ND	ND
0.01	R009	ND	ND	R057	ND	ND
0.01	R010	ND	ND	R058	ND	ND
0.01	R011	ND	ND	R059	ND	ND
0.01	R012	ND	ND	R060	ND	ND
0.01	R013	ND	ND	R061	ND	ND
0.1	R014	ND	ND	R062	ND	ND
0.1	R015	ND	ND	R063	ND	ND
0.1	R016	ND	ND	R064	ND	ND
0.1	R017	ND	ND	R065	ND	ND
0.1	R018	ND	ND	R066	ND	ND
0.3	R019	2.31	5.77	R067	ND	ND
0.3	R020	1.47	3.68	R068	ND	ND
0.3	R021	0.381	0.95‡	R069	ND	ND
0.3	R022	0.921	2.30	R070	ND	ND
0.3	R023	0.340	0.85‡	R071	ND	ND
1	R024	1.34	3.34	R072	ND	ND
1	R025	2.23	5.56	R073	ND	ND
1	R026	2.73	6.81	R074	ND	ND
1	R027	2.38	5.95	R075	ND	ND
1	R028	2.73	6.81	R076	ND	ND
3	R029	3.10	7.74	R077	ND	ND
3	R030	4.61	11.5	R078	ND	ND
3	R031	5.83	14.6	R079	ND	ND
3	R032	4.92	12.3	R080	ND	ND
3	R033	3.19	7.97	R081	ND	ND
10	R034	7.94	19.9	R082	0.301	0.753‡
10	R035	8.96	22.4	R083	0.776	1.94
10	R036	6.84	17.1	R084	0.700	1.75
10	R037	9.88	24.7	R085	1.18	2.96
10	R038	7.36	18.4	R086	1.13	2.81
30	R039	14.5	36.2	R087	1.72	4.31
30	R040	16.3	40.7	R088	2.56	6.39
30	R041	15.3	38.2	R089	1.26	3.15
30	R042	15.2	38.3	R090	1.35	3.37
30	R043	24.0	60.1	R091	2.38	5.96
100	R044	67.1	168	R092	1.66	4.16
100	R045	88.3	221	R093	0.885	2.21
100	R046	41.9	105	R094	4.07	10.2
100	R047	34.0	85.1	R095	2.32	5.79
100	R048	44.5	5.77	R096	0.826	2.07

ND: Analyte not detected above the Limit of Quantitation (LOQ): 1.73 μM.
‡Concentration estimated based on half the LOQ.

Table S15. Individual PFHxSA liver-plasma partitioning coefficients (Kp)

Dose Level (mg/kg/day)	Male Rat ID	Partition Coefficient (K_p)	Female Rat ID	Partition Coefficient (K_p)
0	R001	N/A	R049	N/A
0	R002	N/A	R050	N/A
0	R003	N/A	R051	N/A
0	R004	N/A	R052	N/A
0	R005	N/A	R053	N/A
0	R006	N/A	R054	N/A
0	R007	N/A	R055	N/A
0	R008	N/A	R056	N/A
0.01	R009	N/A	R057	N/A
0.01	R010	N/A	R058	N/A
0.01	R011	N/A	R059	N/A
0.01	R012	N/A	R060	N/A
0.01	R013	N/A	R061	N/A
0.1	R014	0.662	R062	0.424
0.1	R015	0.433	R063	0.516
0.1	R016	0.319	R064	0.491
0.1	R017	0.860	R065	0.293
0.1	R018	0.744	R066	0.378
0.3	R019	0.362	R067	0.633
0.3	R020	0.134	R068	0.239
0.3	R021	0.315	R069	0.486
0.3	R022	0.255	R070	0.499
0.3	R023	0.215	R071	0.302
1	R024	0.166	R072	0.197
1	R025	0.233	R073	0.224
1	R026	0.174	R074	0.122
1	R027	0.293	R075	0.183
1	R028	0.249	R076	0.325
3	R029	0.193	R077	0.158
3	R030	0.153	R078	0.280
3	R031	0.235	R079	0.189
3	R032	0.105	R080	0.239
3	R033	0.244	R081	0.244
10	R034	0.191	R082	0.183
10	R035	0.249	R083	0.145
10	R036	0.178	R084	0.119
10	R037	0.194	R085	0.113
10	R038	0.135	R086	0.113
30	R039	0.156	R087	0.154
30	R040	0.135	R088	0.134
30	R041	0.142	R089	0.142
30	R042	0.145	R090	0.162
30	R043	0.142	R091	0.147
100	R044	0.506	R092	0.187
100	R045	0.754	R093	0.154
100	R046	0.306	R094	0.344
100	R047	0.230	R095	0.214
100	R048	0.221	R096	0.213

N/A: Not applicable.
